# Reference population for international comparisons and time trend surveillance of preterm delivery proportions in three countries

**DOI:** 10.1186/1472-6874-8-16

**Published:** 2008-09-25

**Authors:** Nils-Halvdan Morken, Ida Vogel, Karin Kallen, Rolv Skjærven, Jens Langhoff-Roos, Ulrik Schiøler Kesmodel, Bo Jacobsson

**Affiliations:** 1 Department of Obstetrics and Gynecology, Telemark Hospital, Skien, Norway; 2 Perinatal Center, Department of Obstetrics and Gynecology, Institute for the Health of Women and Children, Sahlgrenska Academy, Göteborg, Sweden; 3 Norwegian Institute of Public Health, Oslo, Norway; 4 NANEA at the Institute of Public Health, Department of Epidemiology, University of Aarhus, Denmark; 5 Department of Clinical Genetics, Aarhus University Hospital, Aarhus, Denmark; 6 Tornblad Institute, University of Lund, Lund, Sweden; 7 Section for Epidemiology and Medical Statistics, University of Bergen, Bergen, Norway; 8 Medical Birth Registry of Norway, Norwegian Institute of Public Health, Bergen, Norway; 9 Department of Obstetrics, Rigshospitalet, Copenhagen, Denmark

## Abstract

**Background:**

International comparison and time trend surveillance of preterm delivery rates is complex. New techniques that could facilitate interpretation of such rates are needed.

**Methods:**

We studied all live births and stillbirths (≥ 28 weeks gestation) registered in the medical birth registers in Sweden, Denmark and Norway from 1995 through 2004. Gestational age was determined by best estimate. A reference population of pregnant women was designed using the following criteria: 1) maternal age 20–35, 2) primiparity, 3) spontaneously conceived pregnancy, 4) singleton pregnancy and 5) mother born in the respective country. National preterm delivery rate, preterm delivery rate in the reference population and rate of spontaneous preterm delivery in the reference population were calculated for each country.

**Results:**

The total national preterm delivery rate (< 37 completed gestational weeks), increased in both Denmark (5.3% to 6.1%, p < 0.001) and Norway (6.0% to 6.4%, p = 0.006), but remained unchanged in Sweden, during 1995–2004. In Denmark, the preterm delivery rate in the reference population (5.3% to 6.3%, p < 0.001) and the spontaneous preterm delivery rate in the reference population (4.4% to 6.8%, p < 0.001) increased significantly. No similar increase was evident in Norway. In Sweden, rates in the reference population remained stable.

**Conclusion:**

Reference populations can facilitate overview and thereby explanations for changing preterm delivery rates. The model also permits comparisons over time. This model may in its simplicity prove to be a valuable supplement to assessments of national preterm delivery rates for public health surveillance.

## Background

International comparison of common complex disease incidences and prevalences are difficult, and no common standard on how to perform such comparisons exist. Several risk factors contribute to a certain common complex trait, but the same factors may have different implications in different populations, resulting in often incomparable crude rates between nations, regions and over time. In obstetrics, preterm delivery rates are reported to be very different, ranging from 5–20%. It is obvious that this to some extent is due to different levels of well known risk factors and contributors of preterm delivery, such as artificial reproductive treatment, multiple births, ethnic admixture and varying extent to which preterm gestations are induced.

Preterm delivery is a serious global health problem and one of the leading causes of child death worldwide [1]. International comparisons of preterm delivery rates are complex, mainly due to the heterogeneity of the preterm populations. Core questions such as "Does the baseline preterm delivery rate differ between populations?" or "Does the preterm delivery rate increase over time?" are difficult to answer due to the number of factors influencing this rate. Different ways of registering gestational age and the use of either pregnancies or children as the observational unit [2], as well as ethnicity and obstetric and socio-demographic factors, influence numbers and make comparisons challenging [3]. The frequency of several of these risk factors, well known for their association with preterm delivery, also changes over time and differs between countries. Assisted reproduction, high maternal age, primiparity and elective delivery before term have changed in recent years, changes that have been used to explain the increase in preterm delivery rates seen in several countries [4-6]. Better living conditions, declines in smoking and specific prevention programs may have contributed to the decreased preterm delivery rates seen in a few other countries [7-9].

The reported incidences of preterm delivery in the international literature vary considerably between countries and also between ethnic groups within countries [10]. The three Scandinavian countries are similar in terms of antenatal care and socio-demographic factors and they have homogeneous populations that derive from a common Nordic ancestry. Despite this, there are differences in the preterm delivery rates. Denmark has reported a remarkable increase (27%) during the last decade [11]. Norway has also reported an increase, from 7.5% in 1995 to 8.5% in 2002 [12], whereas Sweden is one of few countries in the world that has reported a decrease in the preterm delivery rate from 6.3% in 1984 to 5.6% in 2001 [7].

We have previously applied the concept of studying a reference population and defined a group of low-risk primiparous women (Caucasian, 20–40 years of age, with singleton spontaneous pregnancies and spontaneous onset of delivery) in a Danish cohort [11]. The idea was that this simple restriction into a reference population would enable direct comparison of preterm delivery rates and spontaneous preterm delivery rates from different countries per se, without the use of complex statistical techniques as in confounder analyses.

In the current report we use preterm delivery rate as an application of the concept of studying reference populations for international comparison, but we believe that this very simple model can be applied in studies of other complex traits and in other medical disciplines. We aimed to compare data from the three Scandinavian birth registers in Sweden, Denmark, and Norway, respectively, during 1995 through 2004.

## Methods

A population-based multinational comparative study was conducted. All live births and stillbirths (≥ 28 weeks) registered from 1995 to 2004 in the medical birth registers in Sweden, Denmark and Norway, were included. These birth registers collect data prospectively, starting at the first antenatal visit. All three data sources contain information on birth weight, gestational age, smoking habits (beginning in 1999 in Norway) and socio-demographic factors as well as complications during pregnancy, delivery and neonatal period.

The Swedish Medical Birth Register is validated annually against the National Population Register using the mother's and the infant's unique personal identification numbers, and contains data on more than 99% of all births. A quality analysis of the register has previously been conducted [13]. In Denmark, linkage of four national registers (the National Patient Register, the Civil Registration System Register, the IVF Register and the Cause of Death Register), using the mother's unique personal identification number, constitutes the Danish Medical Birth Register. A quality analysis of all births registered during one day has been conducted [14]. All records in the Medical Birth Registry of Norway are matched with the files of the Central Person Registry, ensuring medical registration of every newborn in Norway, and the register contains information on very close to all births in the country [15].

A reference population of pregnant women was designed using the following criteria: 1) maternal age 20–35 at delivery, 2) primiparity, 3) spontaneously conceived pregnancy, i. e. excluding assisted reproduction, 4) singleton pregnancy and 5) woman born in the respective country studied (citizenship at childbirth was used when country of birth was not available). The latter criterion was included to account for differences in rates over time caused by immigration.

In Sweden, there are four bases for calculating gestational age: date of last menstrual period, corrected expected date of parturition according to last menstrual period (corrected according to the length of the menstrual cycle), expected date of parturition according to ultrasound and the estimated gestational age at birth reported by the delivery unit. Using these sources hierarchically, as described in detail elsewhere [16], the best available estimate of gestational age for each infant is determined and designated best estimate. In Denmark, the designated best clinical estimate of gestational age is based on information from obstetric records (including last menstrual period and ultrasound data) and is reported to the National Patient Register immediately after delivery. In Norway, gestational age determination was based on last menstrual period through 1998. Beginning in 1999, ultrasound-based estimation of gestational age has also been registered. In the case of Norway, we decided to use last menstrual period only in the absence of gestational age determination by ultrasound. We decided to let best estimates for each individual country determine gestational age in this study and cases with unknown gestational age were excluded. To avoid obvious misclassifications of gestational age, deliveries were excluded if the infants' birth weight and length exceeded 4 standard deviations according to national growth curves [17,18].

Preterm delivery was defined as delivery occurring between 22 weeks and 0 days and 36 weeks and 6 days of gestation. It was further categorized into: extreme (22 to 27 gestational weeks), very (28 to 31 gestational weeks) and moderate (32 to 36 gestational weeks) preterm delivery. Onset of labor was registered in all three countries as spontaneous, including preterm labor and preterm prelabor rupture of membranes, or iatrogenic, including induced labor or caesarean section. The two latter groups were regarded as iatrogenic preterm births in the present study. In cases of preterm pre-labor rupture of the membranes (ICD 9: 6581; ICD 10: O42), births were regarded as spontaneous preterm births regardless of the reported onset of labor. The onset of labor variable in The Swedish Medical Birth Register has been validated in a small pilot study based on previously published data [19] and was found to be reliable [16].

Time trends were assessed using simple logistic regression analyses with dichotomized variables as outcome variables and year of birth as the only independent variable. P-values < 0.05 were considered statistically significant. To test for homogeneity of the ORs across the three local estimates (OR_i_), the log (OR_i_*): s were weighted according to precision to compute a pooled estimate (OR_m_): OR_m_* = exp(Σ(w_i _(log(OR_i_*))/Σw_i_), w_i _= 1/V(log(OR_i_*)), and the test statistics used was X_i _= (Σ(w_i _(log(OR_i_*) - log(OR_m_*))^2^, which was compared to the χ^2^(2) – distribution.

The ethics committee in Sweden approved the study. In Denmark and Norway register-based studies are exempt from such approval.

## Results

The total population undergoing analysis constituted 2 111 014 deliveries of which 912 227 (43.2%) were Swedish, 648 388 (30.7%) were Danish and 550 399 (26.1%) were Norwegian. Less than 1.5% of the total number of registered deliveries in the three Scandinavian countries from 1995 to 2004 was excluded due to missing gestational age or obvious misclassification.

Preterm delivery rates, proportions of known risk factors and proportions of the reference populations in the respective national figures are presented in Tables 1 to 3, according to gestational age and year of birth for each country. The total preterm delivery rate (< 37 completed gestational weeks) increased in both Denmark (p < 0.001) and Norway (p = 0.006), but was unchanged in Sweden over the decade. The proportions of the different risk factors changed over time, but the tendencies were rather similar in the three countries, except an unchanged proportion of multiple births in Sweden (p = 0.48), while significant increases occurred in both Denmark and Norway (p < 0.001). The proportion of the reference population in the national population increased significantly in Sweden (p < 0.001) during this decade, while decreasing in Denmark and Norway (p < 0.001).

**Table 1:** The proportion of preterm deliveries in Sweden, 1995–2004, presented according to gestational age groups, selected risk factors and the rate and absolute number of the reference population.

**Sweden**	**1995**	**1996**	**1997**	**1998**	**1999**	**2000**	**2001**	**2002**	**2003**	**2004**	**p-value for trend^4^**
**Deliveries (n)**	100319	93104	87451	84243	84473	87710	88634	92041	95740	98512	
**22–27 w**	0.22	0.24	0.22	0.28	0.29	0.21	0.26	0.27	0.29	0.26	0.012 (I)^5^
**28–31 w**	0.56	0.63	0.57	0.58	0.61	0.58	0.60	0.62	0.58	0.57	0.63
**32–36 w**	4.9	4.8	4.8	4.8	4.9	4.9	4.8	4.8	4.8	5.0	0.91
**< 37 w**	5.6	5.7	5.6	5.7	5.8	5.7	5.6	5.7	5.7	5.8	0.41

**Women not born in Sweden**	17.5	17.7	18.0	18.3	18.2	18.2	18.5	18.8	19.0	19.8	< 0.001 (I)
**IVF or ICSI**	0.6	1.4	1.8	1.6	1.8	1.6	1.8	2.0	2.2	2.2	< 0.001 (I)
**Multiples**	1.5	1.5	1.6	1.6	1.6	1.5	1.6	1.5	1.7	1.4	0.48
**Primiparous^1^**	40.2	41.3	41.4	41.5	42.3	44.0	44.7	45.5	45.2	45.0	< 0.001 (I)
**Iatrogenic delivery^2^**	14.1	14.0	14.9	15.9	17.0	16.9	18.1	18.5	19.0	19.5	< 0.001 (I)
**Age < 20**	2.1	2.0	2.0	1.9	2.0	2.0	1.9	1.9	1.7	1.6	< 0.001(R)^6^
**Age ≥35**	13.3	13.8	14.1	15.4	16.0	16.8	17.9	18.3	19.3	19.9	< 0.001 (I)
											
**Reference population^3 ^(% of all births)**	29.6	29.9	30.0	29.6	29.9	31.2	31.3	31.7	31.1	30.3	< 0.001 (I)

**Reference population^3 ^(n)**	29685	27875	26249	24926	25232	27354	27762	29133	29775	29825	

P-values for trend are presented.

^1 ^Primiparous: the index pregnancy resulted in delivery for the first time.

^2 ^Induced delivery or caesarean section.

^3 ^Reference population: 20 – 35-years-old primiparas, born in Sweden, with singleton, spontaneously conceived pregnancies.

^4 ^p-values obtained from simple logistic regression analyses with the dichotomized variables specified as outcome variables, and year of birth as the only independent variable.

^5^(I) = Increase

^6^(R) = Reduction

**Table 2:** The proportion of preterm deliveries in Denmark, 1995–2004, presented according to gestational age groups, selected risk factors and the rate and absolute number of the reference population.

**Denmark**	**1995**	**1996**	**1997**	**1998**	**1999**	**2000**	**2001**	**2002**	**2003**	**2004**	**p-value for trend^4^**
**Deliveries (n)**	68081	65891	66041	64816	64769	65699	64014	62648	63203	63226	
**22–27 w**	0.22	0.26	0.22	0.20	0.23	0.21	0.26	0.23	0.29	0.25	< 0.001(I)^5^
**28–31 w**	0.60	0.68	0.63	0.65	0.64	0.65	0.63	0.65	0.67	0.67	0.11
**32–36 w**	4.5	4.9	4.5	4.7	4.6	4.8	5.2	5.2	5.2	5.2	< 0.001(I)
**< 37 w**	5.3	5.9	5.3	5.5	5.5	5.7	6.0	6.1	6.2	6.1	< 0.001(I)

**Women not born in Denmark**	17.0	18.3	19.2	19.6	20.4	20.8	20.9	21.2	20.8	20.2	< 0.001(I)
**IVF or ICSI**	1.2	1.5	1.9	2.0	2.4	2.8	3.0	3.0	3.4	2.5	< 0.001 (I)
**Multiples**	1.7	1.9	1.9	2.0	2.0	2.0	2.2	2.2	2.2	2.3	< 0.001(I)
**Primiparous^1^**	44.9	45.7	43.0	43.0	43.3	43.8	43.9	43.5	43.7	43.9	< 0.001(R)^6^
**Iatrogenic delivery^2^**	15.0	15.3	10.0	11.5	13.7	15.6	17.3	19.4	19.8	20.7	< 0.001(I)
**Age < 20**	2.0	1.9	1.9	1.8	1.7	1.6	1.6	1.4	1.3	1.3	< 0.001(R)
**Age ≥35**	11.4	11.9	12.7	13.3	14.2	14.7	15.7	16.9	17.3	17.3	< 0.001(I)
											
**Reference population^3 ^(% of all births)**	33.3	33.4	30.3	30.4	30.1	30.2	30.2	29.6	29.8	30.6	< 0.001(R)

**Reference population^3 ^(n)**	22699	21977	20000	19732	19460	19843	19338	18522	18856	19322	

P-values for trend are presented.

^1 ^Primiparous: the index pregnancy resulted in delivery for the first time.

^2 ^Induced delivery or caesarean section.

^3 ^Reference population: 20 – 35-years-old primiparas, born in Denmark, with singleton, spontaneously conceived pregnancies.

^4 ^p-values obtained from simple logistic regression analyses with the dichotomized variables specified as outcome variables, and year of birth as the only independent variable.

^5^(I) = Increase

^6^(R) = Reduction

**Table 3:** The proportion of preterm deliveries in Norway, 1995–2004, presented according to gestational age groups, selected risk factors and the rate and absolute number of the reference population.

**Norway**	**1995**	**1996**	**1997**	**1998**	**1999**	**2000**	**2001**	**2002**	**2003**	**2004**	**p-value for trend^4^**
**Deliveries (n)**	53044	54058	53023	52113	58098	58024	55519	54545	55791	56184	
**22–27 w**	0.22	0.23	0.23	0.25	0.28	0.32	0.29	0.32	0.31	0.26	< 0.001(I)^5^
**28–31 w**	0.64	0.67	0.61	0.68	0.73	0.70	0.68	0.70	0.74	0.63	0.14
**32–36 w**	5.1	4.9	5.1	5.1	5.1	5.2	5.4	5.5	5.2	5.5	< 0.001(I)
**< 37 w**	6.0	5.8	5.9	6.0	6.2	6.2	6.4	6.5	6.2	6.4	< 0.006(I)

**Women not born in Norway**	9.9	10.2	10.7	11.4	12.4	13.7	13.9	15.1	16.0	16.5	< 0.001(I)
**IVF or ICSI**	0.7	0.7	0.8	1.0	1.4	1.5	1.5	1.7	1.8	1.9	< 0.001 (I)
**Multiples**	1.6	1.5	1.6	1.6	1.8	1.8	1.8	1.9	1.9	1.9	< 0.001(I)
**Primiparous^1^**	40.9	40.9	40.4	41.0	42.9	41.0	40.9	41.1	41.6	42.2	< 0.001(I)
**Iatrogenic delivery^2^**	15.9	16.1	15.8	15.2	16.6	16.1	17.1	17.0	18.0	18.5	< 0.001(I)
**Age < 20**	2.7	2.7	2.6	2.6	2.6	2.6	2.6	2.5	2.2	2.0	< 0.001(R)^6^
**Age ≥35**	12.0	12.2	13.1	13.5	13.6	14.2	15.1	15.4	16.4	17.0	< 0.001(I)
											
**Reference population^3 ^(% of all births)**	32.0	32.1	31.3	31.1	31.9	30.0	29.7	29.3	29.3	29.7	< 0.001(R)

**Reference population^3 ^(n)**	16971	17354	16619	16202	18516	17423	16511	15972	16331	16675	

P-values for trend are presented.

^1 ^Primiparous: the index pregnancy resulted in delivery for the first time.

^2 ^Induced delivery or caesarean section.

^3 ^Reference population: 20 – 35-years-old primiparas, born in Norway, with singleton, spontaneously conceived pregnancies.

^4 ^p-values obtained from simple logistic regression analyses with the dichotomized variables specified as outcome variables, and year of birth as the only independent variable.

^5^(I) = Increase

^6^(R) = Reduction

The reference population in each country is described in detail in Table 4. The proportion of preterm delivery according to gestational age, country and year of birth are presented. In Denmark, the preterm delivery rate in the reference population increased significantly (p < 0.001). This increase was evident among moderate (32–36 weeks) preterm deliveries. In Sweden, the rate of preterm delivery in the reference population was unchanged, while there was a small, but significant (p = 0.018), reduction in Norway. The test for homogeneity of trends shows that there are differences between the three countries in terms of the preterm delivery rate in the reference population, evident at gestational age 32–36 weeks (p < 0.001) and 28–31 weeks (p = 0.0034).

**Table 4:** The proportion of preterm deliveries in the reference population^1 ^in Sweden (S), Denmark (D) and Norway (N), 1995–2004, presented according to gestational age groups.

**Gestational weeks**	**Country**	**1995**	**1996**	**1997**	**1998**	**1999**	**2000**	**2001**	**2002**	**2003**	**2004**	**p-value for trend^2^**
**22–27**	S	0.18	0.22	0.22	0.22	0.27	0.15	0.19	0.20	0.25	0.26	0.13
	**D**	0.19	0.22	0.23	0.19	0.20	0.19	0.22	0.13	0.27	0.21	0.83
	**N**	0.18	0.20	0.21	0.20	0.15	0.27	0.18	0.20	0.19	0.14	0.65
p for homogeneity of trends: 0.37												
**28–31**	**S**	0.55	0.69	0.56	0.60	0.64	0.54	0.62	0.62	0.59	0.57	0.80
	**D**	0.66	0.67	0.62	0.67	0.65	0.57	0.61	0.63	0.59	0.66	0.48
	**N**	0.53	0.43	0.40	0.61	0.38	0.34	0.35	0.45	0.35	0.26	< 0.001(R)^3^
p for homogeneity of trends: 0.0034												
**32–36**	**S**	5.5	5.3	5.1	5.2	5.4	5.5	5.0	5.3	5.2	5.6	0.82
	**D**	4.5	5.0	4.3	4.8	5.0	5.0	5.2	5.4	5.6	5.4	< 0.001(I)^4^
	**N**	4.7	4.5	4.6	5.1	4.3	4.4	4.6	4.7	4.4	4.4	0.18
p for homogeneity of trends: < 0.001												
**< 37**	**S**	6.3	6.2	5.9	6.0	6.3	6.2	5.9	6.1	6.1	6.5	0.67
	**D**	5.3	5.9	5.2	5.6	5.9	5.7	6.0	6.2	6.5	6.3	< 0.001(I)
	**N**	5.4	5.1	5.2	5.9	4.8	5.0	5.2	5.3	5.0	4.8	0.018 (R)
p for homogeneity of trends: < 0.001												

^1 ^Reference population: 20 – 35-years-old primiparas, born in the country in which delivery occurs, with singleton, spontaneously conceived pregnancies.

^2 ^p-values obtained from simple logistic regression analyses with the dichotomized variables specified as outcome variables, and year of birth as the only independent variable.

^3^(R) = Reduction

^4^(I) = Increase

Rates of *spontaneous *preterm delivery compared to the total number of deliveries in the reference population are presented according to gestational age, country and year of birth in Table 5. The Danish spontaneous preterm delivery rate increased significantly (p < 0.001) from 1995 to 2004. In Sweden, the incidence was unchanged (p = 0.64). In Norway there was a major change in registration in 1999, which is clearly visible in the figures. From 1999 to 2004, the rate of spontaneous preterm deliveries in the reference population was unchanged (p = 0.63). If trends are tested from 1995 to 2004, there was a significant reduction.

**Table 5:** The proportion of spontaneous preterm deliveries of the total number of deliveries in the reference population^1 ^in Sweden (S), Denmark (D) and Norway (N), 1995–2004, presented according to gestational age groups.

**Gestational weeks**	**Country**	**1995**	**1996**	**1997**	**1998**	**1999**	**2000**	**2001**	**2002**	**2003**	**2004**	**p-value for trend^2^**
**22–27**	**S**	0.10	0.13	0.13	0.11	0.11	0.07	0.12	0.11	0.12	0.16	0.34
	**D**	0.17	0.18	0.23	0.21	0.23	0.21	0.23	0.13	0.31	0.24	< 0.038(I)^3^
	**N**	0.15	0.17	0.17	0.17	0.13	0.23	0.15	0.15	0.16	0.12	0.33^4^
												
**28–31**	**S**	0.22	0.26	0.24	0.25	0.28	0.19	0.23	0.24	0.28	0.27	0.44
	**D**	0.45	0.44	0.58	0.64	0.71	0.58	0.63	0.71	0.63	0.72	< 0.001(I)
	**N**	0.44	0.36	0.34	0.52	0.30	0.29	0.30	0.36	0.29	0.22	0.98^4^
												
**32–36**	**S**	4.1	3.8	3.5	3.6	3.7	3.9	3.4	3.7	3.7	3.9	0.39
	**D**	3.8	4.4	4.3	4.8	5.2	5.2	5.2	5.7	5.8	5.8	< 0.001(I)
	**N**	4.0	3.8	3.9	4.4	3.6	3.7	3.9	3.9	3.7	3.7	0.29^4^
												
**< 37**	**S**	4.4	4.2	3.9	4.0	4.1	4.1	3.8	4.0	4.1	4.3	0.64
	**D**	4.4	5.0	5.1	5.7	6.1	6.0	6.0	6.5	6.7	6.8	< 0.001(I)
	**N**	4.6	4.3	4.4	5.0	4.0	4.2	4.3	4.5	4.2	4.0	0.63^4^

^1 ^Reference population: 20 – 35-years-old primiparas, born in the country in which delivery occurs, with singleton, spontaneously conceived pregnancies.

^2 ^p-values obtained from simple logistic regression analyses with the dichotomized variables specified as outcome variables, and year of birth as the only independent variable for 1995 to 2004.

^3^(I) = Increase

^4^Trend analyzed for 1999 to 2004, due to known registration changes in the Medical Birth Register of Norway in 1999.

## Discussion

The design and use of reference populations when analyzing Scandinavian birth register data show that changes in maternal age, parity, assisted reproduction, multiple births and immigration can explain the increase in preterm birth in countries such as Norway. However, these factors cannot explain the increase in Denmark and no trend was found in the Swedish data.

By utilizing reference populations as a supplement to the more general surveillance methods, reporting national crude preterm delivery proportions can elucidate that the traditional and well known risk factors don't have the impact in a population as previously believed, such as in the case with Denmark. This can open up the search for new risk factors. Furthermore, the use of reference populations gives the opportunity to perform more specific surveillance of low-risk patients and the most important and least well understood subgroup of preterm delivery, the spontaneous onset births. The proposed method is clearly meant as a supplement to reporting of national crude preterm delivery proportions and especially well designed for easy international comparison.

The model using a reference population was applied to control for known confounding from major risk factors that vary over time and between countries. The factors used to identify the reference population were selected from a combination of clinical experience, availability and literature reviews of risk factors for preterm delivery. It might, for instance, be argued that smokers should be eliminated from the reference population as smoking is relatively common and increases the risk of preterm delivery. However, registration of smoking is often not valid or even omitted. Inclusion of such factors would decrease the validity of data and the number of countries from which data could be obtained and was thus rejected. Calculation of the national rates as well as the rate in the reference population can provide answers to several questions that would otherwise require complex statistics, i.e. "Is there a basic rate of preterm delivery?" "Has it changed over time?" "Can the factors eliminated in the reference population explain changes across populations or over time?" The suggested model can thus facilitate meaningful international comparison. The omission of smoking clearly illustrates that this model cannot replace more complex analyses such as logistic regression analyses.

The complexity of the entity under study and the fact that the data used in this study was from three secondary data sources (The three Scandinavian birth registers), resulted in that the final decisions made about which criteria that should be used to define the reference population to a great extent were compromises. This kind of data limited our access to some variables, as is the case in terms of information related to previous induced abortion and late second trimester abortions. However, we believe that we have decided upon the criteria for the reference population in a way that optimizes the ability to use this sub-population for international comparison.

Increasing ethnic diversity, increasing contribution from assisted reproduction, increasing number of multiple births, increasing maternal age and increasing relative number of iatrogenic preterm deliveries have all been used previously as arguments for increasing preterm delivery rates [4-6,20]. Primiparity was reduced in Denmark, which should have contributed to a decreasing preterm delivery rate. Multiple pregnancies increased in both Denmark and Norway, while remaining unaltered in Sweden. Despite rather consistent changes in risk factors among the Scandinavian countries, clear differences were evident in terms of time trends for the proportion of spontaneous preterm deliveries in the reference population. In our opinion, this indicates that current understanding of different risk factors and their respective contributions to the changing preterm delivery rate is incomplete.

The recent temporal increase in the overall preterm delivery rate in both the United States and France is said to be driven by an impressive concomitant increase in medically indicated preterm deliveries [4,21]. Our data shows an increasing number of iatrogenic preterm deliveries in all three Scandinavian countries, which should contribute to an increased total preterm delivery rate. Furthermore, according to the literature, the increasing incidence of multiple pregnancies associated with assisted reproduction is another main cause of increased preterm delivery rates [4,22-24]. The availability and use of these techniques have increased in all three countries. However, neither medically indicated nor multiple preterm deliveries can be the only explanation for the increased rate of spontaneous preterm delivery found in the Danish reference population, as was argued in previous reports, which illustrates the benefit of using this method. It is therefore clear that the medically indicated and multiple preterm deliveries cannot be the only explanation for increased preterm delivery rates, as argued by previous reports.

It is well known that gestational age determination by ultrasound increases the incidence of preterm delivery [6]. Might not the increase in preterm delivery rates in Denmark and Norway just be the result of an increased use of ultrasound? Gestational age determination by ultrasound has, however, been extensively used during the whole study period and there has been no recent change in clinical guidelines in any of the three countries. A Danish study of a cohort from 1990–1999 reported that the reduction in gestational age during the study period occurred regardless of whether the calculation was based on the last menstrual period or on ultrasound [25]. It thus seems that there has been a true decrease in gestational age over the study period.

A factor not accounted for in the current study is smoking. Smoking during pregnancy is a well known risk factor for preterm delivery and one that is reducible. Smoking was not registered in The Medical Birth Registry of Norway until 1999. There has been a substantial decrease in smoking during pregnancy in all three countries during the study period [7,11,26]. These figures are similar in the three Scandinavian countries and should most likely have contributed to a decreased preterm delivery rate; they can therefore not explain the diversities found.

The increased spontaneous preterm delivery rate found in the Danish reference population is a cause for concern and most certainly a true and reliable finding. Several possible explanations exist: increased/altered genital infections, increased maternal stress during pregnancy, changed dietary intake, increased occurrence of chronic maternal disease during pregnancy, altered clinical management and changes and deterioration in antenatal care. The current study cannot answer this question; future studies are thus required.

Total preterm delivery rates, based on crude national figures, do not necessarily provide overview of development over time. This is illustrated by the Norwegian national crude data indicating an increased preterm delivery problem, while findings in the reference population gave no reason for concern. In Denmark, the increasing preterm delivery rate was also seen in the reference population, including among the spontaneous preterm deliveries. Using reference populations in the assessment and comparison of preterm delivery rates is simple and may in the future prove useful at the local, national and international level as it facilitates interpretation without the use of complex statistics. This method may thus yield more adequate time trend analyses, facilitating international comparison.

## Conclusion

Reference populations can facilitate overview and thereby explanations for changing preterm delivery rates. The model also permits comparisons over time. This model may in its simplicity prove to be a valuable supplement to assessments of national preterm delivery rates for public health surveillance.

## Competing interests

The authors declare that they have no competing interests.

## Authors' contributions

All investigators of this study had full access to all of the data in the study and take responsibility for the integrity of the data and the accuracy of the data analysis. BJ, IV, JLR and USK were responsible for formulating the hypothesis, contributing to the study design and commenting on manuscript drafts. KK and RS did the searches in the data bases, contributed to the study design, did the raw analysis of data and commented on report drafts. NHM has contributed to the study design, been responsible for interpretation of data analysis and drafted the manuscript.

## Pre-publication history

The pre-publication history for this paper can be accessed here:



## References

[B1] Bryce J, Boschi-Pinto C, Shibuya K, Black RE (2005). WHO estimates of the causes of death in children. Lancet.

[B2] Lumley J (1993). The epidemiology of preterm birth. Baillieres Clin Obstet Gynaecol.

[B3] Savitz DA, Blackmore CA, Thorp JM (1991). Epidemiologic characteristics of preterm delivery: etiologic heterogeneity. Am J Obstet Gynecol.

[B4] Papiernik E, Zeitlin J, Rivera L, Bucourt M, Topuz B (2003). Preterm birth in a French population: the importance of births by medical decision. Bjog.

[B5] Joseph KS, Kramer MS (1999). Recent versus historical trends in preterm birth in Canada. Cmaj.

[B6] Joseph KS, Kramer MS, Marcoux S, Ohlsson A, Wen SW, Allen A, Platt R (1998). Determinants of preterm birth rates in Canada from 1981 through 1983 and from 1992 through 1994. N Engl J Med.

[B7] Morken NH, Kallen K, Hagberg H, Jacobsson B (2005). Preterm birth in Sweden 1973–2001: Rate, subgroups, and effect of changing patterns in multiple births, maternal age, and smoking. Acta Obstet Gynecol Scand.

[B8] Olsen P, Laara E, Rantakallio P, Jarvelin MR, Sarpola A, Hartikainen AL (1995). Epidemiology of preterm delivery in two birth cohorts with an interval of 20 years. Am J Epidemiol.

[B9] Papiernik E, Bouyer J, Dreyfus J, Collin D, Winisdorffer G, Guegen S, Lecomte M, Lazar P (1985). Prevention of preterm births: a perinatal study in Haguenau, France. Pediatrics.

[B10] Slattery MM, Morrison JJ (2002). Preterm delivery. Lancet.

[B11] Langhoff-Roos J, Kesmodel U, Jacobsson B, Rasmussen S, Vogel I (2006). Spontaneous preterm delivery in primiparous women at low risk in Denmark: population based study. Bmj.

[B12] (2004). Medical Birth Registry of Norway Annual Report.

[B13] Cnattingius S, Ericson A, Gunnarskog J, Kallen B (1990). A quality study of a medical birth registry. Scand J Soc Med.

[B14] Langhoff-Roos JRS (2003). [Validering af Landspatientregisteret (LPR) med henblik på obstetrisk forskning og kvalitetssikring].

[B15] Irgens LM (2000). The Medical Birth Registry of Norway. Epidemiological research and surveillance throughout 30 years. Acta Obstet Gynecol Scand.

[B16] Morken NH, Kallen K, Jacobsson B (2006). Fetal growth and onset of delivery: a nationwide population-based study of preterm infants. Am J Obstet Gynecol.

[B17] Marsal K, Persson PH, Larsen T, Lilja H, Selbing A, Sultan B (1996). Intrauterine growth curves based on ultrasonically estimated foetal weights. Acta Paediatr.

[B18] Skjaerven R, Gjessing HK, Bakketeig LS (2000). Birthweight by gestational age in Norway. Acta Obstet Gynecol Scand.

[B19] Jacobsson B, Hagberg G, Hagberg B, Ladfors L, Niklasson A, Hagberg H (2002). Cerebral palsy in preterm infants: a population-based case-control study of antenatal and intrapartal risk factors. Acta Paediatr.

[B20] Papiernik E (1999). Fetal growth retardation: a limit for the further reduction of preterm births. Matern Child Health J.

[B21] Ananth CV, Vintzileos AM (2006). Epidemiology of preterm birth and its clinical subtypes. J Matern Fetal Neonatal Med.

[B22] Joseph KS, Allen AC, Dodds L, Vincer MJ, Armson BA (2001). Causes and consequences of recent increases in preterm birth among twins. Obstet Gynecol.

[B23] Steer P (2006). The epidemiology of preterm labour-why have advances not equated to reduced incidence?. Bjog.

[B24] Smulian JC, Ananth CV, Kinzler WL, Kontopoulos E, Vintzileos AM (2004). Twin deliveries in the United States over three decades: an age-period-cohort analysis. Obstet Gynecol.

[B25] Orskou J, Kesmodel U, Henriksen TB, Secher NJ (2001). An increasing proportion of infants weigh more than 4000 grams at birth. Acta Obstet Gynecol Scand.

[B26] Haug K, Irgens LM, Skjaerven R, Markestad T, Baste V, Schreuder P (2000). Maternal smoking and birthweight: effect modification of period, maternal age and paternal smoking. Acta Obstet Gynecol Scand.

